# Supplementary material for: A study protocol for predictors of post-discharge mortality among children aged 5–14 years admitted to tertiary hospitals in Tanzania: A prospective observational cohort study
Source: PLoS One. 2024 May 22;19(5):e0287243. doi: 10.1371/journal.pone.0287243 (PMC11111011; doi:10.1371/journal.pone.0287243)
Supplement: Attachment — Submitted filename: Response to reviewers.docx [file pone.0287243.s001.docx]

| S/N | Comment(s) | Response(s) | Reference page |
| --- | --- | --- | --- |
| Editors’ comments | |  |  |
| 1 | The protocol lacks research question or hypothesis which would provide focused direction of the study | The research questions have been added to the protocol | Page 4 |
| 2 | While the candidate variables for predicting post-discharge mortality are listed, there is no explicit explanation of their rationale or theoretical basis | A text has been added before each tables with variables | Page 7 and 8 |
| 3 | The author has not provided details about data collection approach: How the questionnaire will be administered and how the research assistants will be trained. | This has been explained well | Page 9 |
| 4 | In the data analysis section, it has not been mention how confounders will be addressed. How will the missing data be addressed | This has been explained in the data analysis | Page 11 |
| 5 | The discussion section should expand to explain the expected results, the limitations and how to mitigate them | This has been explained in the last paragraphs of the discussion | Page 13 |
| 6 | Why was it necessary to conduct the study in the three institutions? | This has been explained in the methodology | Page 5 |
| 7 | Clarify about HINARI | This has been responded | Page 15 |
| 8 | There is inconsistencies in the methodology, in some sentence it is written participants will be recruited conveniently and in the other consecutively | This has been corrected and participants will be recruited conveniently before discharge | Page 6 |
| 9 | Explain well sample size calculation | This has been explained well | Page 6 |
| 10 | Grammar | Grammar mistakes have been checked using grammarly app |  |
| 11 | Abbreviations not in alphabetical order | Put in alphabetical order | Page 14 |
| 12 | There is controversy about the funding | This has been corrected | Page 14 |
